# Ammonia induced microglia activation was associated with limited effects on connexin 43 and aquaporin 4 expression in an astrocyte-microglia co-culture model

**DOI:** 10.1186/s12868-021-00628-1

**Published:** 2021-03-25

**Authors:** Fatme Seval Ismail, Timo Jendrik Faustmann, Franco Corvace, Anamariya Tsvetanova, Zahra Moinfar, Pedro M. Faustmann

**Affiliations:** 1 Department of Neurology, University Hospital Knappschaftskrankenhaus Bochum, Ruhr University Bochum, Bochum, Germany; 2 Department of Psychiatry and Psychotherapy, Medical Faculty, Heinrich Heine University, Düsseldorf, Germany; 3 Department of Neuroanatomy and Molecular Brain Research, Ruhr University Bochum, Bochum, Germany; 4 International Graduate School of Neuroscience, Ruhr University Bochum, Bochum, Germany

**Keywords:** Ammonia, Astrocyte-microglia co-culture model, Microglial activation, Connexin 43, Aquaporin 4

## Abstract

**Background:**

Hepatic encephalopathy (HE) is a neurological complication resulting from acute or chronic liver disease. Hyperammonemia leading to astrocyte swelling and cerebral edema in combination with neuroinflammation including microglia activation, mainly contribute to the pathogenesis of HE. However, little is known about microglia and their inflammatory response, as well as their influence on astrocytic channels and astrocyte swelling under hyperammonemia.

**Objective:**

To investigate the effects of ammonia on the microglial activation and morphology in different set-ups of an in vitro astrocyte-microglia co-culture model. Further, potential effects on glial viability, connexin 43 (Cx43) and aquaporin 4 (AQP4) expression were tested.

**Methods:**

Primary rat glial co-cultures of astrocytes containing 5% (M5, representing "physiological" conditions) or 30% (M30, representing "pathological" conditions) of microglia were incubated with 3 mM, 5 mM, 10 mM and 20 mM ammonium chloride (NH4Cl) for 6 h and 24 h in order to mimic the conditions of HE. An MTT assay was performed to measure the viability, proliferation and cytotoxicity of cells. The microglial phenotypes were analyzed by immunocytochemistry. The expression of Cx43 and AQP4 were quantified by immunoblot analysis.

**Results:**

A significant reduction of glial viability was observed in M30 co-cultures after incubation with 20 mM NH4Cl for 6 h, whereas in M5 co-cultures the viability remained unchanged. Microglial activation was detected by immunocytochemistry after incubation with 3 mM, 5 mM and 10 mM NH4Cl for 6 h and 24 h in M5 as well as in M30 co-cultures. The Cx43 expression was slightly increased in M30 co-cultures after 6 h incubation with 5 mM NH4Cl. Also, the AQP4 expression was slightly increased only in M5 co-cultures treated with 10 mM NH4Cl for 6 h. Under the other conditions, Cx43 and AQP4 expression was not affected by NH4Cl.

**Conclusions:**

The novel aspect of our study was the significant microglial activation and decrease of viability after NH4Cl incubation in different set-ups of an in vitro astrocyte-microglia co-culture model, contributing to better understanding of pathophysiological mechanisms of HE. Hyperammonemia led to limited effects on Cx43 and AQP4 expression, the relevance of these minimal changes should be viewed with caution.

**Supplementary Information:**

The online version contains supplementary material available at 10.1186/s12868-021-00628-1.

## Background

Hepatic encephalopathy (HE) is a neurological complication resulting from acute or chronic liver disease. High ammonia level resulting from impaired liver function can cross the blood–brain-barrier and lead to morphological changes of astrocytes [1, 2]. Further, astrocytes are the main cells in the central nervous system (CNS) which can metabolize ammonia into glutamine using glutamine synthetase [3, 4]. Elevated ammonia and glutamine level in astrocytes results in increased water accumulation, cytotoxic astrocyte swelling and increased osmotic pressure contributing to cerebral edema, and correlates with the state of HE [5–8]. Studies have also shown that the transmembrane water channel protein Aquaporin 4 (AQP4), which is localized on astrocyte end-feet and regulates maintaining brain water homeostasis, is up-regulated during acute liver failure and HE [9–14].

Microglia, the main immune cells of the CNS, are found in a resting ramified form in the healthy brain and range from 5 to 20% of the glial cell population [15]. Under pathological conditions, microglial activation includes proliferation of microglia, change of the morphological phenotype from the resting ramified type (RRT) to the activated, rounded phagocytic type (RPT), expression of immune molecules and release of inflammatory mediators [16]. Intermediate type of microglia (INT) represents the phenotypic transition from resting ramified to the activated type and are characterized by short cell processes. Studies have demonstrated that hyperammonemia in HE leads to microglia activation in terms of stimulation of microglial cell migration, morphological changes, oxidative stress and up-regulation of the microglial activation marker ionized calcium-binding adaptor molecule-1 (Iba-1) [17]. Media from ammonia-treated microglia cell culture added to cultured astrocytes contributes to astrocyte swelling suggesting a link between astrocytes and microglia under hyperammonemia conditions [18].

In summary, hyperammonemia leading to astrocyte swelling and cerebral edema in combination with neuroinflammation including microglia activation, mainly contribute to the pathogenesis of HE.

Connexin 43 (Cx43) is the predominant protein in astrocytes that contributes to formation of gap junctions (GJs) and functional astrocytic network allowing the exchange of small molecules, ions and second messengers, and ensuring the homeostasis [19]. Connexin 43 is further involved in spatial buffering of potassium, cell proliferation, regulation of transmitter uptake and dissipation, support of neurons, and volume regulation [15, 19]. There is evidence that gap-junctional coupling of astrocytes plays a role in ammonia-induced cytotoxicity [20]. No data about effects of ammonia on Cx43 expression in astrocytes are available to date. Also, little is known about microglia and their influence on astrocytic channels and astrocyte swelling under hyperammonemia. In this study, we aimed to investigate the effects of ammonia on the microglial activation and morphology in different set-ups of an in vitro astrocyte-microglia co-culture model. Further, potential effects on glial viability, connexin 43 (Cx43) and aquaporin 4 (AQP4) expression were tested.

## Methods

### Cell culture

Primary astrocyte-microglia co-cultures were derived from brain of postnatal Wistar rats (postnatal day 0–2, P0–P2) according to Faustmann et al. [15]. All experiments were performed according to the German animal welfare act and the ethical standards of Ruhr University Bochum, and were approved by the local authorities in Bochum, Germany. All animals were kept under standard laboratory conditions with access to food and water.

The P0–P2 rats were decapitated without sedation, according to the German animal welfare act. After removing of cerebellum, meninges and choroid plexus, the brains were kept in ice-cold phosphatebuffered saline (PBS) (containing 1,38 M NaCl, 27 mM KCl, 81 mM NaH2PO4, 14,7 mM K2H2PO4 (J.T. Baker, Deventer, the Netherlands), then treated with 0.1% trypsin (PAA laboratories, Pasching, Austria) for 30 min at 37 °C and centrifuged at 500×*g* for 12 min to remove the supernatant. Following this, the pellet was resuspended in 5 ml of DNase I solution (Serva Electrophoresis, Heidelberg, Germany) (100 µl/ml with Dulbecco’s minimal essential medium, DMEM, Invitrogen, Karlsruhe, Germany) for 5 min at room temperature, centrifuged at 200×*g* for 5 min and after washing steps (washing medium containing 10% FCS (Biochrom AG, Berlin, Germany), 1% penicilin/ streptomycin solution (PAA Laboratories, Linz, Austria)) filtered through a 60-μm nylon mesh. Cells were kept at a density of one brain per plastic tissue-culture flask in 7% CO2 at 37 °C in astrocyte culture medium (containing 10% fetal calf serum, 1% non-essential amino acids, 1% glutamine, 1% penicilin/streptomycin solution) (PAA Laboratories, Linz, Austria). After 5 days, the cultures were about 100% confluent. Adherent microglial cells and oligodendroglia on the astroglial surface were separated from the culture by shaking the flasks manually. The amount of microglial cells in the co-cultures varied between 5 and 30% depending on the extent of shaking and was determined by counting after fixation and staining.

### Treatment of cultures

The primary rat glial co-cultures of astrocytes containing 5–10% (M5, representing "physiological" conditions) or 30–40% (M30, representing "pathological" conditions) of microglia were incubated with 3 mM, 5 mM, 10 mM and 20 mM ammonium chloride (NH4Cl) (diluted in H2O) (Sigma-Aldrich, Germany) for 6 h and 24 h in 7% CO_2_ at 37 °C in order to mimic the conditions of HE. Because the survival of the glial cells after incubation with high concentration (20 mM) of NH4Cl can be affected, it was not used for experiments testing microglia morphology, Cx43 and AQP4 expression.

### MTT assay

An MTT (3-(4,5-dimethylthiazol-2-yl)-2,5-diphenyltetrazolium bromide) assay (Roche applied sciences) was performed to measure the viability, proliferation and cytotoxicity of cells. The co-cultures from tissue-culture flask were placed on poly-l-lysine-coated glass cover slips at 12,000 cells per well in 94-well plates in 7% CO_2_ at 37 °C until they were confluent. Cells were incubated with ammonium chloride as described above. In the next step, incubation with 10 µl MTT reagent for 4 h in 7% CO2 at 37 °C was performed. Following this, 100 µl of solubilization solution were applied to the co-cultures and the samples were incubated overnight. The next day, the Bio-rad microplate reader (München, Germany) was used to measure the cell viability in the wells at a wavelength of 550 nm.

### Immunocytochemistry

**Fig. 1:**
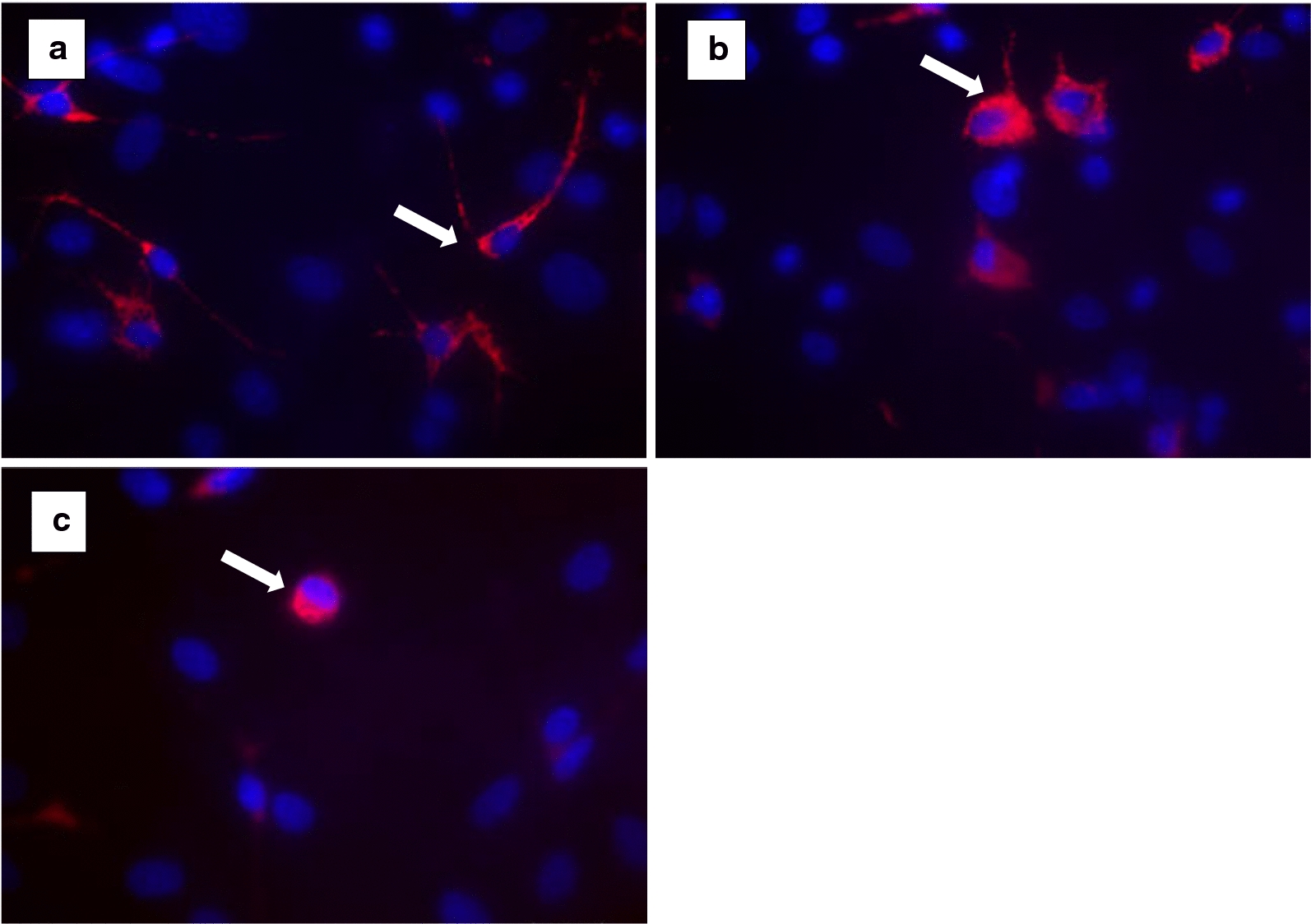
Microglia morphology (*white arrows*) at a magnification of 630 × . ED1 staining allowed the classification of microglia as resting ramified (**a**), intermediate (**b**) and activated rounded phagocytic (**c**) phenotype under hyperammonemia

The microglial phenotypes as well as Cx43 and AQP4 expression were analyzed by immunocytochemistry. The astrocyte-microglia co-cultures were placed on poly-l-lysine-coated glass cover slips at 70,000 cells per well in 24-well plates and incubated with ammonium chloride as described above. Cover slips with the cell cultures were fixed with 70% ethanol for 10 min and incubated in PBS-blocking solution containing 1% bovine serum albumin (1% BSA) (PAA Laboratories, Linz, Austria). The cover slips were treated with rabbit anti-Cx43 (1:2000) (Invitrogen, Karlsbad, Germany) and rabbit anti-AQP4 (1:200) (Invitrogen, Karlsbad, Germany) in combination with mouse anti-ED1 (1:250) (Serotec, Düsseldorf, Germany) (diluted in 1% BSA in PBS) and incubated at 4 °C for 2 h. In the next step, the wells were incubated with secondary antibodies (1:500) (diluted in 1% BSA, 10% horse serum in PBS) (Invitrogen, Karlsruhe, Germany) including goat anti-mouse IgG conjugates (Alexa fluor® 568) and goat anti-rabbit IgG conjugates (Alexa fluor® 488) for 1 h. Immunocytochemically labeled cells were counterstained with DAPI (4,6-diamidino-2-phenyl-indol, 1:2500) (Invitrogen, Karlsruhe, Germany) for quantification of cell numbers. The ratio of microglia to astrocytes was identified by comparison of the number of ED1-stained microglia with the total number of DAPI-labeled cells. The microglia morphology was evaluated in a minimum of three different visual fields on each cover slip at a primary magnification of 630 × (total of *n* = 52). ED1 staining allowed the classification of microglia as ramified, intermediate and activated rounded phagocytic phenotype [11] (Fig. 1).

### Immunoblot (western blot) analysis

The expression of Cx43 and AQP4 were quantified by immunoblot analysis according to the protocol. A total number of 300.000 cells were seeded in each culture dish. After reaching confluence, the cells were treated with ammonium chloride as described above. In the next step, the cells were washed with PBS and lysed with 200 μl Laemmli 1 × buffer and 4 μl protease inhibitor cocktail. The cells were detached from the culture dishes using a silicone scraper and the lysates were kept on ice. The protein concentrations were measured by Bradford assay (Bio-rad Bradford Protein Assay, München, Germany) based on the protocol [21]. Next, loading of 10 μg solution onto 10% or 15% sodium dodecyl sulfate (SDS) gel (AppliChem, Darmstadt, Germany) and electrophoresis at 100 V for 20 min and following 150 V was performed. After transfer of the gels to nitrocellulose membrane for Cx43 or polyvinylidene fluoride (PVDF) membrane for AQP4 and blocking with Odyssey blocking buffer (LI-COR Bioscience, Germany) for 1 h, the membranes were incubated with anti-β-actin (1:10,000) (Sigma, St.Louis, USA), anti-Cx43 (1:5000) (Invitrogen, Karlsbad, Germany) or anti-AQP4 (1:2000) (Invitrogen, Karlsbad, Germany) antibodies (diluted in 0,5% blocking buffer) at 4 °C overnight. After washing with 0.1% Tween®20 (AppliChem, Darmstadt, Germany) in PBS (PBST) for 3 × 15 min, the membranes were treated with secondary anti-β-actin peroxidase goat anti-mouse (1:20,000) and peroxidase goat anti-rabbit (1:10,000) fluorescent antibodies (Sigma, St.Louis, USA) (diluted in 0,5% blocking buffer) for 1 h. Washing step with PBST was performed. The Odyssey Infrared Imaging System (LI-COR Bioscience, Germany) was used to visualize the bands. Subsequent quantification of the bands was performed using ImageStudio Lite V5.2 software from LI-COR. The percentage of Cx43 and AQP4 was quantified using Microsoft Excel in ratio to the beta-actin band.

### Data analyses and statistics

All statistical analyses and graphs were performed with GraphPad Prism version 6.0 for Windows (GraphPad Software, San Diego, USA). Normality of data distribution was analyzed using the Kolmogorov–Smirnov tests and D'Agostino-Pearson omnibus tests. Parametric test were only applied when normality was given. Comparisons between more than two groups with normal distribution were analyzed using One-way analysis of variance (One-way ANOVA) and Kruskal–Wallis test. The significance was set at p < 0.05 and the results were reported as mean ± standard error of the mean.

## Results

### Glial viability after incubation with ammonia

**Fig. 2:**
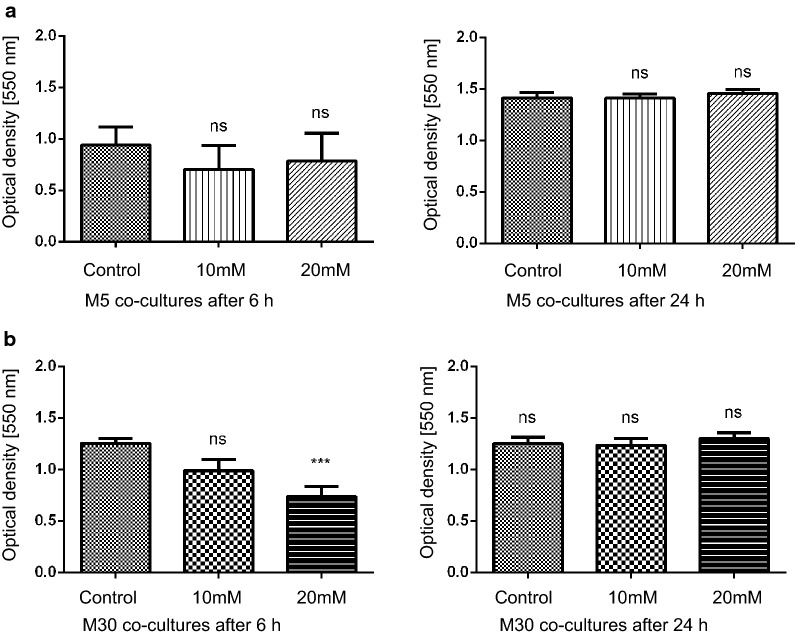
Glial viability detected by MTT assay. **a** In M5 co-cultures, representing „physiological “ conditions, the glial viability was not altered by hyperammonemia. **b** The glial viability was significantly reduced after incubation with 20 mM NH4Cl for 6 h in M30 co-cultures, representing “pathological” conditions (n = 20 independent experiments). This effect could not be detected following NH4Cl incubation for 24 h. Data were collected by n = 8 independent experiments. Comparisons between more than two groups with normal distribution were analyzed using One-way analysis of variance (One-way ANOVA) and Kruskal–Wallis test, *p < 0.05, **p < 0.01, ***p < 0.001

A significant reduction of glial viability was observed after incubation with 20 mM NH4Cl for 6 h in M30 co-cultures, representing „pathological “ conditions (p < 0.001) (Fig. 2b). This effect could not be observed after incubation for 24 h. In M5 co-cultures, representing “physiological” conditions, the glial viability was not altered by hyperammonemia (Fig. 2a).

### Hyperammonemia-induced microglial activation

**Fig. 3:**
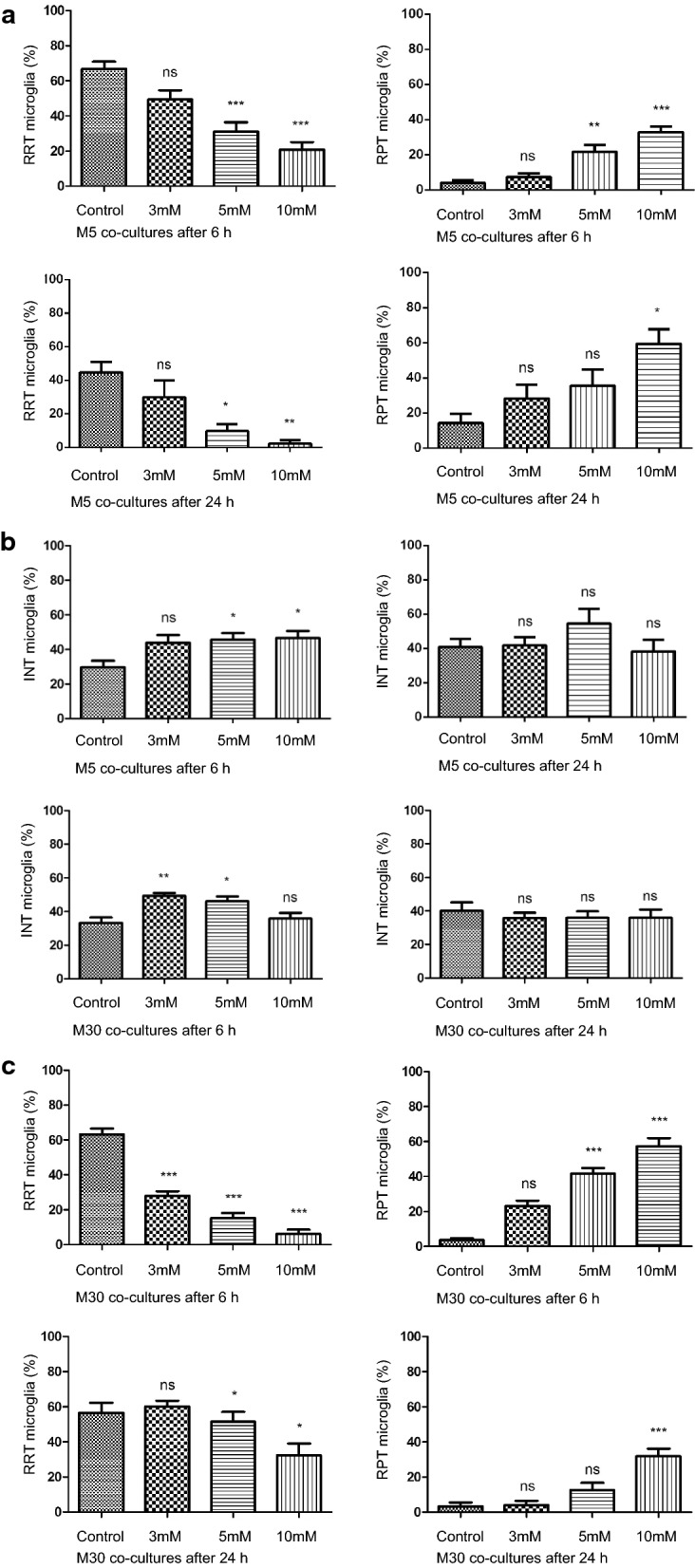
Immunocytochemical analyses of microglial phenotypes under hyperammonemia **a** A significant and dose-dependent increase of activated, rounded phagocytic type of microglia (RPT) was observed in M5 co-cultures by immunocytochemistry after incubation with 5 mM and 10 mM NH4Cl for 6 and 24 h (n = 6 independent experiments). In parallel, the amount of resting ramified type of microglia (RRT) decreased significantly and dose-dependent. **b **The amount of intermediate type of microglia (INT) was increased significantly in M5 co-cultures after incubation with 5 mM and 10 mM NH4Cl for 6 h and in M30 co-cultures after incubation with 3 mM and 5 mM NH4Cl for 6 h. **c **Similar results to **a **were obtained in M30 co-cultures. Data were collected by n = 3 independent experiments. Comparisons between more than two groups with normal distribution were analyzed using One-way analysis of variance (One-way ANOVA) and Kruskal–Wallis test, *p < 0.05, **p < 0.01, ***p < 0.001

We detected a significant and dose-dependent increase of amount of activated microglia in M5 and M30 co-cultures by immunocytochemistry after incubation with 5 mM and 10 mM NH4Cl for 6 and 24 h (Fig. 3a, c). In parallel, the amount of resting microglia decreased significantly and dose-dependent under the same conditions (Fig. 3a, c). Further the amount of intermediate type of microglia was increased significantly in M5 co-cultures after incubation with 5 mM and 10 mM NH4Cl for 6 h (p < 0.05) and in M30 co-cultures after incubation with 3 mM and 5 mM NH4Cl for 6 h (p < 0.01) (Fig. 3b).

### Influence of ammonia on Cx43 expression

**Fig. 4:**
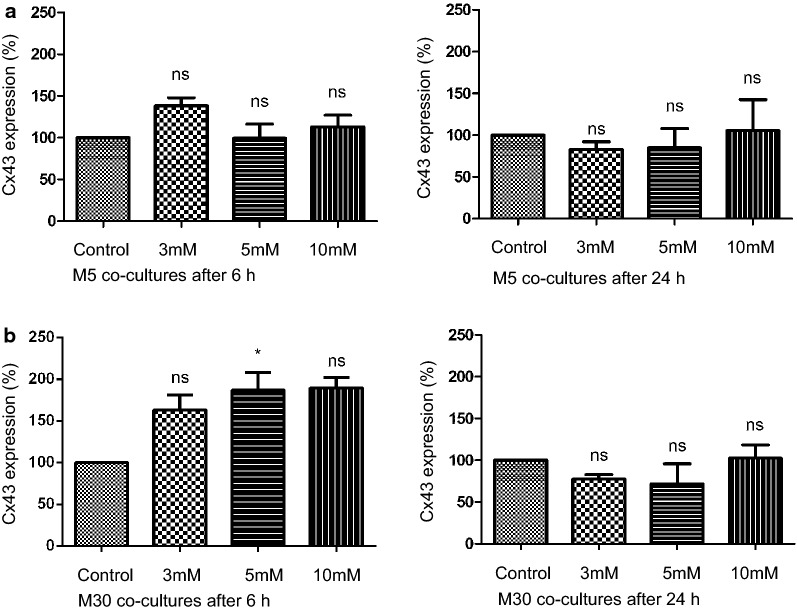
Cx43 expression under hyperammonemia detected by western blot analysis. **a **The Cx43 expression in M5 co-cultures was not altered significantly after incubation with different concentrations of NH4Cl for 6 or 24 h (n = 3 independent experiments). **b** In M30 co-cultures, the Cx43 expression was significantly increased after 6 h incubation with 5 mM NH4Cl and weakly, but not significantly increased after incubation with 10 mM NH4Cl. Incubation for 24 h with NH4Cl did not change the Cx43 expression in the astrocyte-microglia co-cultures. Data were collected by n = 4 independent experiments. Comparisons between more than two groups with normal distribution were analyzed using One-way analysis of variance (One-way ANOVA) and Kruskal–Wallis test, *p < 0.05, **p < 0.01, ***p < 0.001

The Cx43 expression in M5 co-cultures was not changed significantly in immunoblot analysis after incubation with different concentrations of NH4Cl for 6 or 24 h (Fig. 4a). Under “pathological” conditions in M30 co-cultures, the Cx43 expression was significantly increased after 6 h incubation with 5 mM NH4Cl (p < 0.05) and weakly, but not significantly increased after incubation with 10 mM NH4Cl (Figs. 4b and 6a). An additional file shows original, unprocessed versions of full-length representative western blots [see Additional file 1, a]. Longer incubation for 24 h with NH4Cl did not alter the Cx43 expression in the astrocyte-microglia co-cultures.

### Influence of ammonia on AQP4 expression

**Fig. 5:**
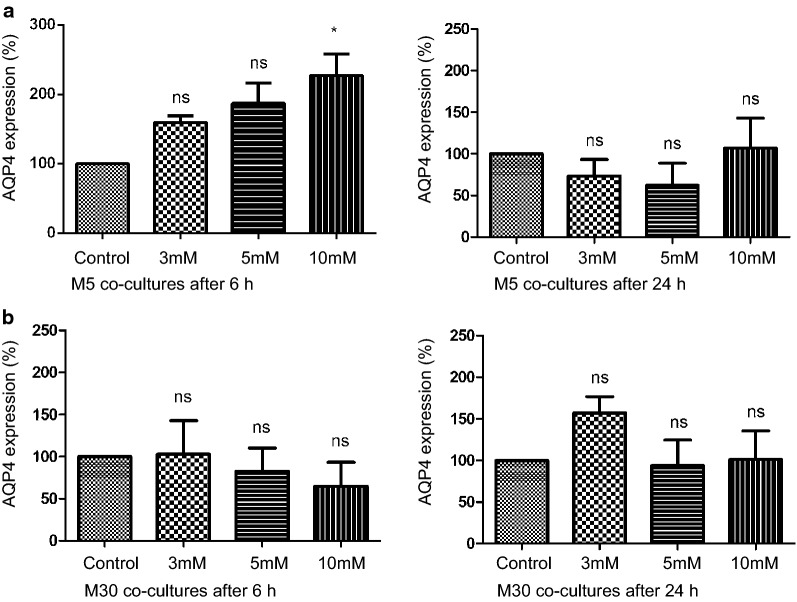
Western blot analysis of AQP4 expression under hyperammonemia. **a** The AQP4 expression was increased significantly only in M5 astrocyte-microglia co-cultures treated with 10 mM NH4Cl for 6 h. This effect was not shown after incubation with NH4Cl for 24 h (n = 4 independent experiments). **b** In M30 co-cultures, AQP4 expression was not altered by ammonia. Data were collected by n = 4 independent experiments. Comparisons between more than two groups with normal distribution were analyzed using One-way analysis of variance (One-way ANOVA) and Kruskal–Wallis test, *p < 0.05, **p < 0.01, ***p < 0.001

**Fig. 6:**
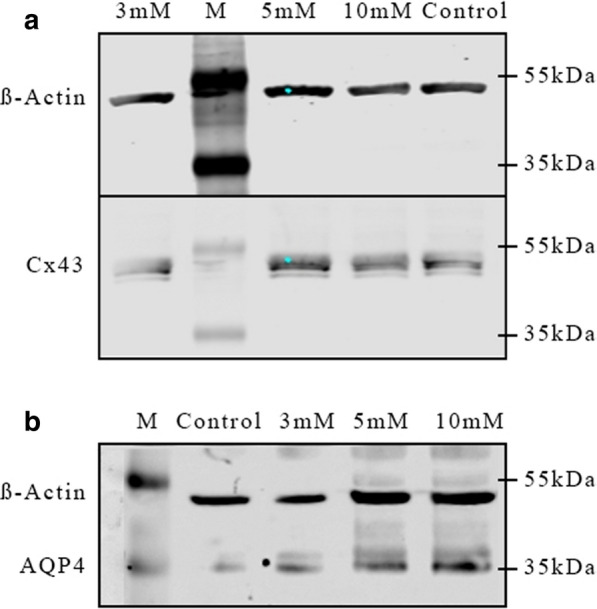
Representative western blots showing the slightly increased Cx43 protein expression (**a**) in M30 co-cultures after 6 h incubation with 5 mM NH4Cl and increased AQP4 expression (**b**) in M5 co-cultures after 6 h incubation with 10 mM NH4Cl

The AQP4 expression was significantly increased only in M5 astrocyte-microglia co-cultures treated with 10 mM NH4Cl for 6 h (p < 0.05) (Figs. 5a and 6b). An additional file shows original, unprocessed versions of full-length representative western blots [see Additional file 1, b]. This effect could not be determined after incubation with NH4Cl for 24 h. In M30 co-cultures, AQP4 expression was not changed after incubation with ammonia (Fig. 5b).

## Discussion

In this study, we demonstrated a significant, dose-dependent increase of microglial activation after incubation with ammonia both in physiological and pathological astrocyte-microglia co-culture model. Ammonia induced limited effects on Cx43 and AQP4 expression under physiological and pathological conditions.

### Glial cell viability under hyperammonemia

Cytotoxic effects of ammonia on primary astrocyte cultures were previously demonstrated, but data about effects on astrocyte-microglia co-culture models were limited available [22, 23]. In our study, we examined the effects of ammonia on glial cell viability in astrocyte-microglia co-culture models under physiological and pathological conditions. A significant reduction of glial viability was observed after incubation with high-dose ammonia for 6 h in M30 astrocyte-microglia co-cultures, representing “pathological” conditions, but not after incubation for 24 h. This could refer to a regulatory effect within the culture after a prolonged time of incubation and could further be explained by a very short half-life of ammonia (1–6.5 s). In M5 co-cultures, representing “physiological” conditions, the glial viability was not affected by hyperammonemia. This effect could not be modulated by changing the concentration of ammonia which underlines how stable and compensatory a physiological culture reacts to stress via ammonia even if the used concentrations was higher than described in previous studies of hepatic encephalopathy [22]. In another study, treatment with “low” concentrations of ammonia (5 mM) for 18 h had also no effects on the cell viability in astroglial-enriched cultures containing up to 10% microglia and in microglia-enriched cultures [24].

### Microglial activation and limited effects on Cx43 and AQP4 expression under hyperammonemia

Our findings showing a significant and dose-dependent increase of activated microglia in M5 and M30 co-cultures after incubation with ammonia, are consistent with previous study results from primary microglia cultures [17, 18]. Incubation of primary microglia cultures with ammonia caused an increase in synthesis and release of IL-6 and TNFα compared to untreated microglia [25]. However, in another study ammonium chloride did not influence LPS-induced up-regulation of transcription of microglia activation markers such as IL-6 and TNFα in microglia mono-cultures, but reduced it in co-cultured astrocytes and microglia [25]. These results indicated that astrocytes reduced the up-regulation of microglia activation markers induced by LPS [25]. Further studies are required to evaluate the exact effects of ammonia on microglia activation in the different contexts of HE.

On the other hand, there is evidence for interactions between astrocytes and microglia under hyperammonemia conditions contributing to astrocyte swelling [18]. Previous in vitro studies by Faustmann et al. [15] demonstrated that microglia activation in M30 astrocyte-microglia co-culture model, representing "pathological" conditions, was associated with reduced astroglial Cx43 expression. However, microglial activation under hyperammonemia was related to slightly increase in Cx43 expression only in one condition in our study. The biological relevance of these minimal and rare significances needs to be questioned. The intercellular coupling in the astroglial network may be modulated by the activation of microglia under pathological conditions in different ways.

The AQP4 expression in our study was sligtly increased after incubation with ammonia only in one condition similar to Cx43. This is consistent with results of previous studies [26]. Further, AQP4 up-regulation after incubation with ammonia correlated with astrocyte swelling [26]. The exact mechanisms leading to increase in AQP4 expression are not yet known. Protein tyrosine nitration was detected in ammonia-treated astrocyte cultures, thus ammonia-induced nitrosative stress may contribute to the up-regulation of AQP4 [27]. It is also conceivable that microglial activation following exposure to pathophysiological concentrations of ammonia can modulate AQP4 expression. However, in our study the ammonia induced microglial activation was associated with a minimal and rare significance in expression of AQP4.

Limitation of our study was that only one method was used for the detection of Cx43 and AQP4 expression under hyperammonemia in the co-culture model, e.g. additional quantitative real-time mRNA expression analysis could be useful and can be focus of future projects. Functional coupling studies of astrocytes and the recording of the astrocytes membrane potential are planned for further investigations [15].

## Conclusions

The novel aspect of our study was the significant microglial activation and decrease of viability after NH4Cl incubation in different set-ups of in vitro astrocyte-microglia co-culture model, contributing to better understanding of pathophysiological mechanisms of HE. Hyperammonemia led to limited effects on Cx43 and AQP4 expression, the relevance of these minimal changes should be viewed with caution. But the astrocyte-microglia co-culture model offers the possibility of investigating the endogenous inflammatory reaction and the cytokine expression under ammonia in a differentiated manner.

## Supplementary Information

**Additional file 1.** Original, unprocessed versions of full-length representative western blots with regard to Fig. 6, showing **(a)** slightly increased Cx43 protein expression in M30 co-cultures after 6 h incubation with 5 mM NH4Cl and increased AQP4 expression **(b)** in M5 co-cultures after 6 h incubation with 10 mM NH4Cl.

## Data Availability

The datasets used or analyzed during this study are available from the corresponding author on reasonable request.

## Abbreviations

AQP4: Aquaporin 4
CNS: Central nervous system
Cx43: Connexin 43
DMEM: Dulbecco’s minimal essential medium
DAPI: 4′,6-Diamidino-2-phenylindole
GJs: Gap junctions
HE: Hepatic encephalopathy
Iba1: Ionized calcium-binding adaptor molecule 1
IL-6: Interleukin 6
INT: Intermediate type
LPS: Lipopolysaccharide
MTT: 3-(4,5-Dimethylthiazol-2-yl)-2,5-diphenyltetrazolium bromide
NH4Cl: Ammonium chloride
PBS: Phosphatebuffered saline
PVDF: Polyvinylidene fluoride
RRT: Resting ramified type
RPT: Rounded phagocytic type
SDS: Sodium dodecyl sulfate
TNFα: Tumor necrosis factor α
