# Supplementary material for: Ammonia induced microglia activation was associated with limited effects on connexin 43 and aquaporin 4 expression in an astrocyte-microglia co-culture model
Source: BMC Neurosci. 2021 Mar 25;22:21. doi: 10.1186/s12868-021-00628-1 (PMC7993489; doi:10.1186/s12868-021-00628-1)
Supplement: Supplementary file 1 — Additional file 1. Original, unprocessed versions of full-length representative western blots with regard to Fig. 6, showing (a) slightly increased Cx43 protein expression in M30 co-cultures after 6 h incubation with 5 mM NH4Cl and increased AQP4 expression (b) in M5 co-cultures after 6 h incubation with 10 mM NH4Cl. [file 12868_2021_628_MOESM1_ESM.docx]

**3mM M 5mM 10mM Control**

**3mM M 5mM 10mM Control**

**a**


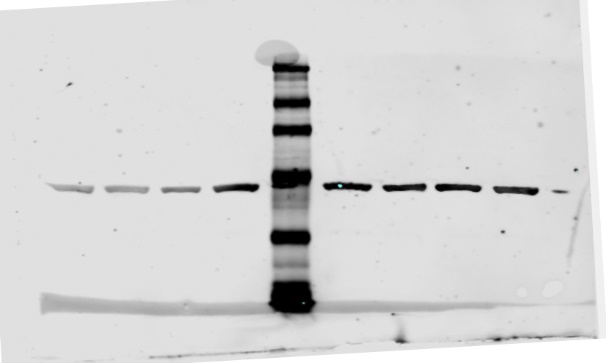

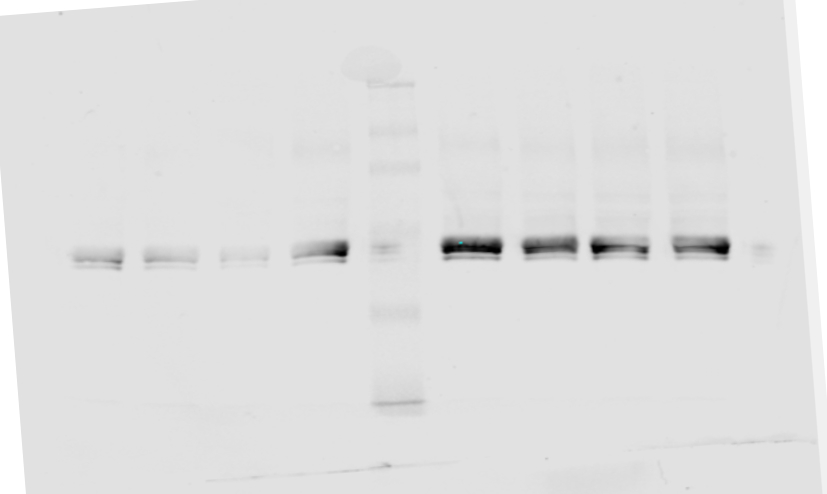


**Cx43** (800nm channel)

**ß-Actin**

(680nm channel)

35 kDa

55 kDa

**b**

**M Control 3mM 5mM 10mM**


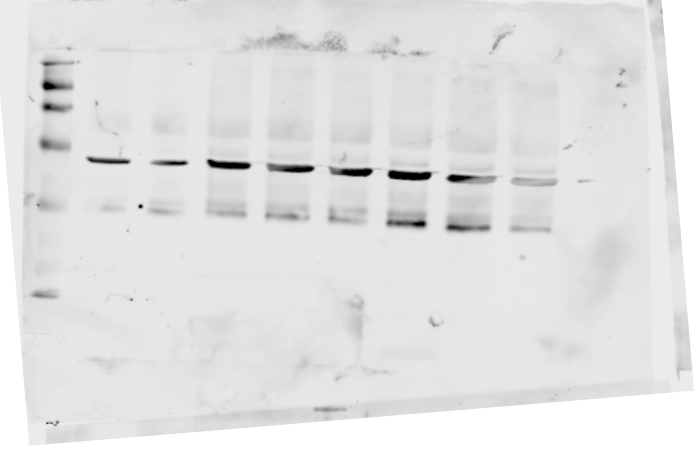


**AQP4**

(800nm channel)

**ß-Actin** (680nm channel)

55 kDa

35 kDa
